# Feature Ranking in Predictive Models for Hospital-Acquired Acute Kidney Injury

**DOI:** 10.1038/s41598-018-35487-0

**Published:** 2018-11-23

**Authors:** Lijuan Wu, Yong Hu, Xiaoxiao Liu, Xiangzhou Zhang, Weiqi Chen, Alan S. L. Yu, John A. Kellum, Lemuel R. Waitman, Mei Liu

**Affiliations:** 1 Big Data Decision Institute (BDDI), Jinan University, Guangzhou, 510632 China; 2 Guangdong Engineering Technology Research Center for Big Data Precision Healthcare, Guangzhou, 510632 China; 3 Division of Nephrology and Hypertension and the Kidney Institute, University of Kansas Medical Center, Kansas City, 66160 USA; 4 Center for Critical Care Nephrology, Department of Critical Care Medicine, University of Pittsburgh School of Medicine, Pittsburgh, 15260 USA; 5 Department of Internal Medicine, Division of Medical Informatics, University of Kansas Medical Center, Kansas City, 66160 USA

## Abstract

Acute Kidney Injury (AKI) is a common complication encountered among hospitalized patients, imposing significantly increased cost, morbidity, and mortality. Early prediction of AKI has profound clinical implications because currently no treatment exists for AKI once it develops. Feature selection (FS) is an essential process for building accurate and interpretable prediction models, but to our best knowledge no study has investigated the robustness and applicability of such selection process for AKI. In this study, we compared eight widely-applied FS methods for AKI prediction using nine-years of electronic medical records (EMR) and examined heterogeneity in feature rankings produced by the methods. FS methods were compared in terms of stability with respect to data sampling variation, similarity between selection results, and AKI prediction performance. Prediction accuracy did not intrinsically guarantee the feature ranking stability. Across different FS methods, the prediction performance did not change significantly, while the importance rankings of features were quite different. A positive correlation was observed between the complexity of suitable FS method and sample size. This study provides several practical implications, including recognizing the importance of feature stability as it is desirable for model reproducibility, identifying important AKI risk factors for further investigation, and facilitating early prediction of AKI.

## Introduction

Acute Kidney Injury (AKI) is a common and highly lethal clinical problem in patients, affecting up to one in five hospitalized adults worldwide^1^. Early prediction or detection of AKI has profound clinical implications but remains a major challenge^2^. Data-driven approaches that incorporate “big” electronic medical record (EMR) data has presented a unique analytic opportunity for AKI, meanwhile a variety of feature selection (FS)^3–5^ methods have been developed to tackle the issue of high dimensionality of EMR data.

Feature selection (FS), including three broad categories^6^: filter, wrapper and embedded methods^3–5^, has become an essential part for developing EMR based predictive models. In AKI predictive modeling, logistic regression with backward or forward selection (wrapper method) is often used to select a subset of features for model building^7^; chi-squared test (filter)^8^, random forest (embedded)^9^, and gradient boosting machine (embedded)^10^ have also been applied to illustrate the feature importance and ranking in AKI prediction. With the increasing variety of feature selection methods and their frequent utilization in the health informatics research community, new questions arise, namely there is no systematic way to choose the most appropriate feature selection method for a given domain and problem, which often depends on two aspects^11^: (a) the stability of FS ranking with respect to different samples, and (b) the prediction accuracy of FS subset effectively representing the entire data. In the context of clinical data analysis, a stable feature selection technique is desirable because selection of relevant clinical risk factors for a given disease on different subsampling of patients should produce similar results. However, most research ignore this aspect and only consider the obtained feature ranking list from a particular method or data sample as a standard and unequivocal result.

To the best of our knowledge, no study has investigated the robustness and applicability of different feature selection techniques and their influence on AKI prediction and risk factor importance ranking. In fact, robustness of feature selection methods has only received attention recently in biomedical applications such as gene and SNP selection, and cancer diagnostics^12,13^. Haury *et al*.^14^ investigated the influence of FS methods on accuracy, stability and interpretability of molecular signatures, and found that the simple filter methods can outperform more complex wrapper or embedded methods. On the contrary, FS methods involving reduced exhaustive search was demonstrated to outperform simple filter methods in another study^15^. The research by Drotár *et al*.^11^ stressed the fact that there is no unique and single solution to the issue of feature selection, and comparative research is important for understanding FS methodology in specific application domains.

In this study, we used nine years of EMR data from a tertiary academic hospital to compare the behavior of eight state-of-the-art FS methods from three aspects: stability of AKI predictor rankings with respect to data sampling variation, similarity between selection results, and AKI prediction performance. The main objective is to investigate which FS method is more suitable for AKI prediction and predictor importance ranking from high-dimensional EMR data.

## Results

**Figure 1:**
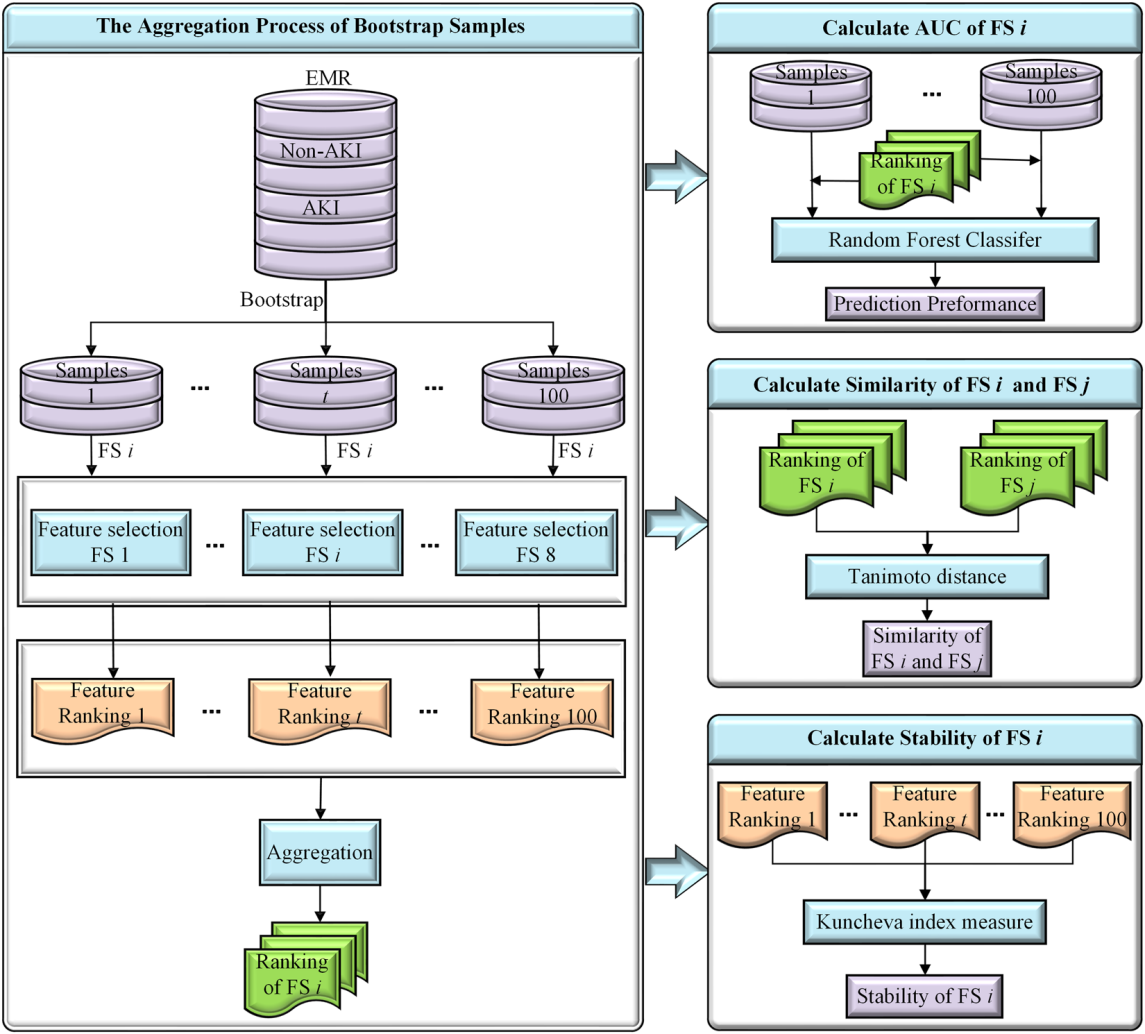
The comparison flow chart of feature selection methods. (*t* denotes the feature ranking of tth bootstrap samples, where 0 < *t* ≤ 100; *i* (or *j*) stands for *i*th (or *j*th) feature selection method, where 1 ≤ *i*, *j* ≤ 8).

The final analysis cohort of the present study consists 76,957 eligible hospital encounters, including all adult patients (age at visit ≥18) who were hospitalized for at least two days from November 2007 to December 2016. Detailed summary of patient demographics in the final analysis cohort is presented in Table 1. In this study, we modeled AKI severity stages separately. Overall AKI occurred in 7,259 (9.43%) encounters with 6,396 (8.31%) at stage 1,678 (0.88%) at stage 2, and 185 (0.24%) at stage 3. Total number of clinical variables collected for each hospital encounter is 1917 (details in Table 2). The flow chart in Fig. 1 illustrates the entire FS comparison experiment conducted in this study.

**Table 1:** Clinical demographics of patients in the analysis cohort.

Characteristic n (%)	AKI-1 (n = 6,396)	AKI-2 (n = 678)	AKI-3 (n = 185)	non-AKI (n = 69,698)	P value
**Age**, **year**					
18–25	303 (4.74)	29 (4.28)	25 (13.51)	4596 (6.59)	<0.001
26–35	514 (8.04)	44 (6.49)	23 (12.43)	7339 (10.53)	<0.001
36–45	711 (11.12)	76 (11.21)	25 (13.51)	8601 (12.34)	0.004
46–55	1218 (19.04)	157 (23.16)	35 (18.92)	14374 (20.62)	0.016
56–64	1672 (26.14)	185 (27.29)	49 (26.49)	16192 (23.23)	<0.001
>64	1978 (30.93)	187 (27.58)	28 (15.14)	18596 (26.68)	<0.001
**Race**					
White	4791 (74.91)	487 (71.83)	130 (70.27)	53177 (76.30)	<0.001
African American	918 (14.35)	111 (16.37)	36 (19.46)	9336 (13.39)	0.003
Asian	45 (0.70)	7 (1.03)	2 (1.08)	600 (0.86)	0.302
Other	642 (10.04)	73 (10.77)	17 (9.19)	6585 (9.45)	0.079
**Gender**					
Male	3822 (59.76)	378 (55.75)	109 (58.92)	37850 (54.31)	<0.001

Note: P value for the comparison of any AKI and non-AKI group was obtained by using Chi-square test.

**Table 2:** Clinical variables considered in the encounters.

Feature Category	# of Variables	Details
Demographics	3	Age, gender, race
Patients’ status	5	BMI, diastolic BP, systolic BP, pulse, temperature
Lab tests	14	Albumin, ALT, AST, Ammonia, Blood Bilirubin, BUN, Ca, CK-MB, CK, Glucose, Lipase, Platelets, Troponin, WBC
Comorbidities	29	University Health System Consortium (UHC) comorbidity
Admission diagnosis	315	University Health System Consortium (UHC) APR-DRG
Medications	1271	All medications are mapped to RxNorm ingredient
Medical History	280	ICD9 codes mapped to CCS major diagnoses

### Feature stability analysis

**Figure 2:**
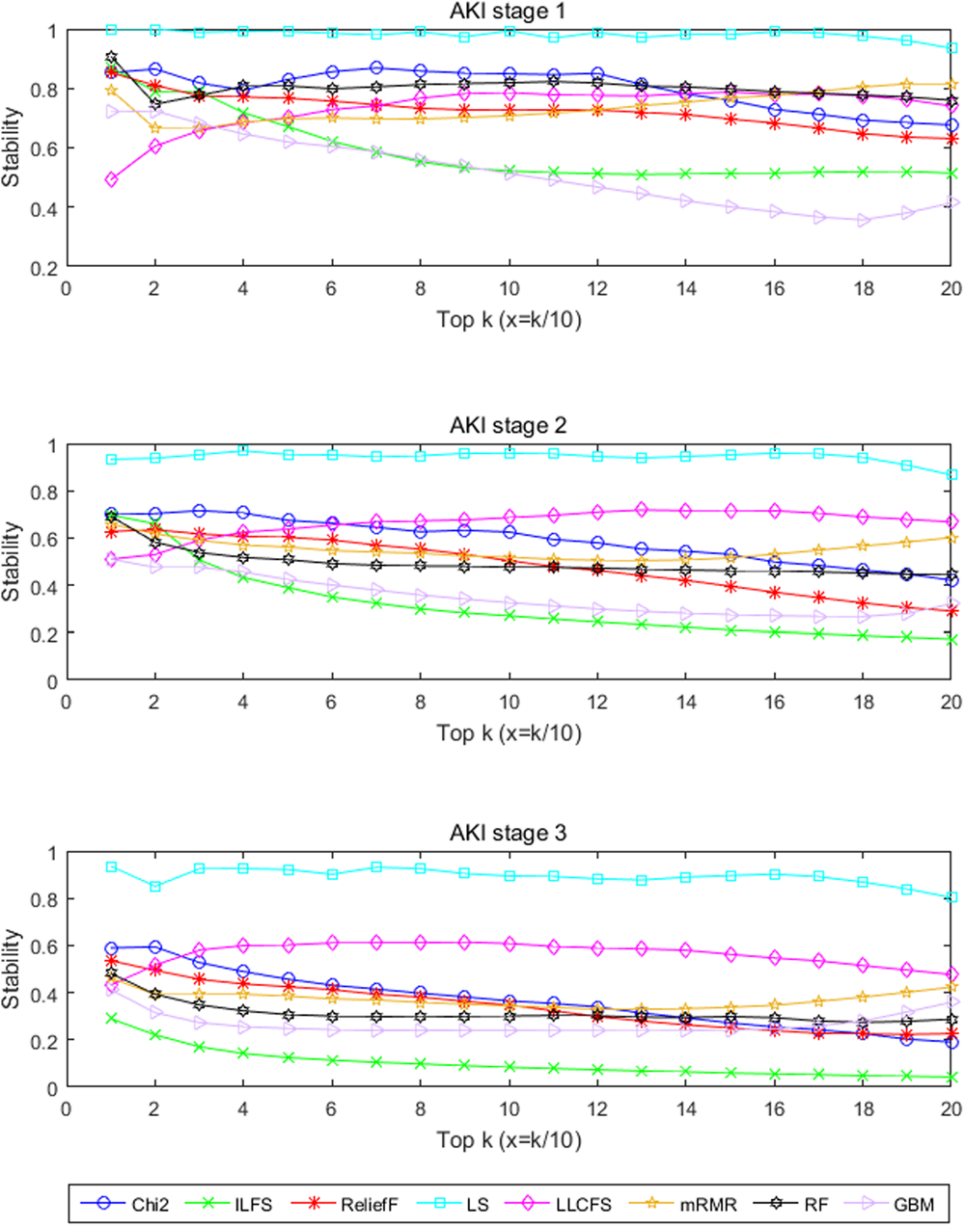
The stability of different feature selection methods.

Stability analysis of each FS method with respect to sampling variation was evaluated as a function of top-*k* (i.e., the number of top-ranking features) where *k* = 10, 20, …, 200 over 100 different bootstrapped samples. Figure 2 shows the stability trend of each FS method with top-*k* (10–200) features for AKI stages 1–3. When comparing the stability results, the unsupervised multivariate filter method Laplacian score (LS) achieved the highest stability values for the AKI datasets.

To obtain a more reliable ranking list based on the same method, aggregation of feature rankings from various bootstrapped data samples was conducted as shown in Fig. 1. Then, we compared the similarity between eight FS methods based on their aggregated rankings. Table 3 compares the similarity of the selected features between eight feature selection methods for AKI stages 1–3. Both multivariate filter methods, e.g. LS and local learning-based clustering with feature selection ILFS (LLCFS), and embedded methods, e.g. gradient boosting machine (GBM) and random forest (RF) algorithms, showed a high degree of similarity. On the contrary, LS and another filter method minimum redundancy-maximum relevance (mRMR) procedure returned the most dissimilar results. Supplementary Table S5 contains details on the top-50 feature sets obtained by eight FS methods, which may serve as basis for further knowledge discovery.

**Table 3:** Similarity of the 8 feature ranking methods with top 50 features.

AKI	Methods	Chi2	ILFS	ReliefF	LS	LLCFS	mRMR	RF	GBM
Stage 1	Chi2	1.00	0.32	0.30	0.22	0.25	0.35	0.28	0.35
	ILFS		1.00	0.39	0.35	0.45	0.25	0.45	0.35
	ReliefF			1.00	0.41	0.45	0.19	0.64	0.39
	LS				1.00	0.59	0.12	0.52	0.25
	LLCFS					1.00	0.22	0.61	0.30
	mRMR						1.00	0.25	0.45
	RF							1.00	0.45
	GBM								1.00
Stage 2	Chi2	1.00	0.32	0.32	0.27	0.30	0.56	0.45	0.39
	ILFS		1.00	0.39	0.35	0.37	0.27	0.39	0.33
	ReliefF			1.00	0.37	0.43	0.27	0.52	0.39
	LS				1.00	0.79	0.19	0.47	0.30
	LLCFS					1.00	0.23	0.52	0.32
	mRMR						1.00	0.37	0.41
	RF							1.00	0.52
	GBM								1.00
Stage 3	Chi2	1.00	0.20	0.25	0.22	0.20	0.54	0.27	0.37
	ILFS		1.00	0.22	0.27	0.28	0.18	0.28	0.28
	ReliefF			1.00	0.30	0.30	0.25	0.32	0.28
	LS				1.00	0.79	0.16	0.45	0.30
	LLCFS					1.00	0.15	0.47	0.32
	mRMR						1.00	0.23	0.33
	RF							1.00	0.56
	GBM								1.00

### Prediction accuracy

**Figure 3:**
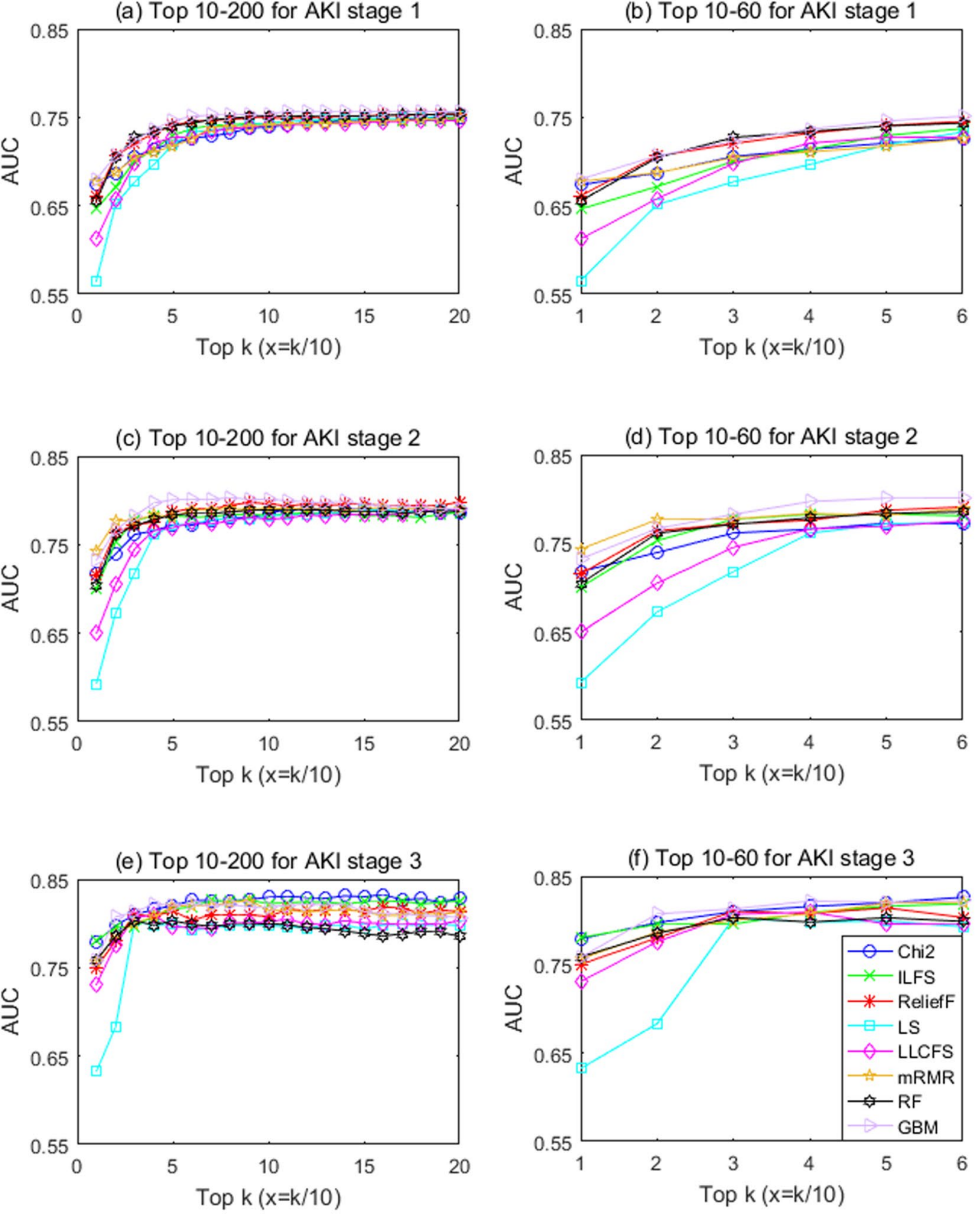
The prediction performance of different feature selection methods.

In terms of prediction performance, as shown in Fig. 3, the area under the receiver operating curve (AUC) increased significantly at the beginning with the increasing number of top features included, and then plateaued around 50. Interestingly for AKI stages 1 and 2 predictions, different feature selection methods converged to a similar AUC after top-200 features were included in the model, while for AKI stage-3 prediction in which much smaller set of samples was available, AUC varied greatly across methods even after top-200 features were included. Among the eight feature selection methods, the complex embedded GBM technique achieved the best prediction performance in most cases. The best AUC for prediction was 0.76 (95% CI, 0.75–0.76) for AKI stage 1, 0.80 (95% CI, 0.80–0.81) for AKI stage 2, and 0.82 (95% CI, 0.81–0.84) for AKI stage 3, respectively. As the AUC increment from additional features across all feature selection methods appear to slow down drastically after top-50 features, this suggest that the minimum feature number required for accurate AKI prediction may be 50.

### Trade-off analysis of stability and accuracy

Although stability matters, stability alone is not a good quality measure because one could conceive a trivial selection algorithm which always returns the same features. We stress the importance of stability as a criterion for choosing an appropriate FS method; however, the selection criteria should not be only based on the stability because a stable ranking is not necessarily accurate. For example, LS performed quite well in terms of stability but achieved rather poor AUC with top-50 features (see Figs 2 and 3), and vice versa, GBM performed well in terms of AKI prediction with top-50 features but achieved rather poor stability (see Figs 2 and 3).

**Figure 4:**
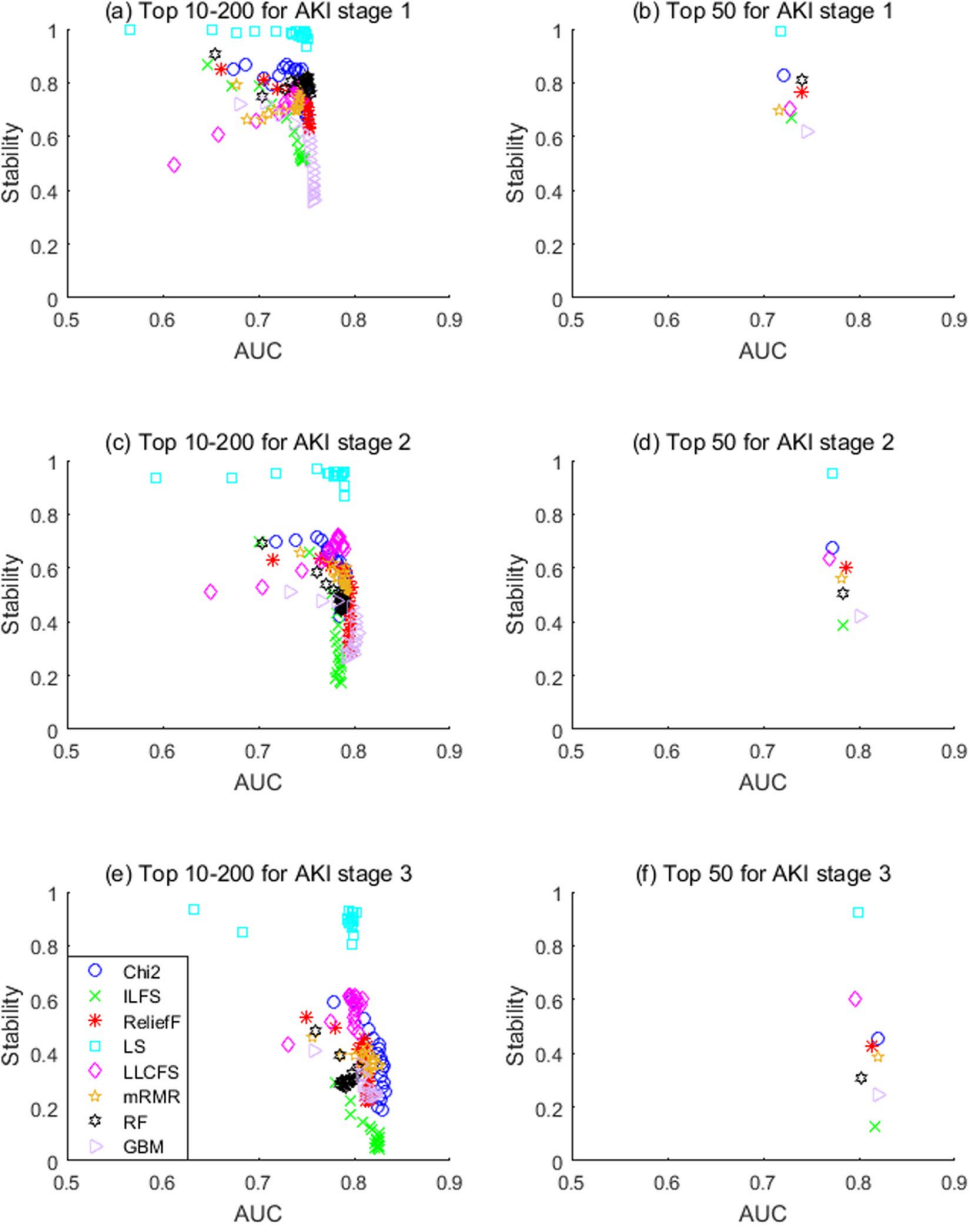
The trade-off between stability and prediction performance.

Hence, others have argued that stability needs to be assessed together with classification performance^16^. Figure 4 illustrates this important trade-off between stability and prediction performance of eight FS methods for each AKI stage. When considering the trade-off between stability and accuracy in choosing an appropriate FS method, we found that the choice depends on the sample size. More specifically, the multivariate embedded RF method appeared to be more suitable for AKI-1 that have larger number of samples, the multivariate filter Relief-F method seemed to be more appropriate for AKI-2 with medium number of samples, and the univariate filter Chi-square test approach was better for small AKI-3 samples.

### Risk factor identification and knowledge discovery

Although different feature selection methods have their own specific criteria for ranking these features, some features are more frequently selected by the methods compared to others. Table 4 shows the top 10 features selected by eight FS methods for AKI stages 1–3, and their corresponding frequency information is illustrated in the Supplementary Table S4. Meanwhile, the top 50 features selected by eight FS methods for AKI stages 1–3 are shown in the Supplementary Table S5. In addition, in Supplementary Table S6, we summarized AKI predictors that not only ranked among the top-50 in this study, but also supported by existing literature.

**Table 4:** Top 10 features selected by 8 feature ranking methods for AKI stages 1–3.

AKI	Chi2	ILFS	ReliefF	LS	LLCFS	mRMR	RF	GBM
Stage 1	MED134	MED746	MED1086	Age	Pulse	MED134	Age	WBC
	MED1086	MED1100	WBC	Temperature	Systolic BP	MED1086	Pulse	MED1086
	MED516	MED582	MED321	Pulse	BMI	WBC	WBC	MED1039
	BUN	MED880	Glucose	WBC	WBC	Glucose	BMI	MED134
	WBC	MED308	MED12	BMI	BUN	MED548	Systolic BP	CCS58
	MED548	Calcium	MED880	Systolic BP	Age	MED1039	MED1086	BUN
	MED746	Glucose	MED134	AST	Glucose	BUN	MED134	COM24
	MED939	MED134	Calcium	BUN	AST	DRG0	BUN	DRG179
	MED321	WBC	Age	Glucose	Bilirubin	DRG3	Calcium	DRG97
	MED880	MED139	MED677	Diastolic BP	Temperature	COM2	MED516	MED319
Stage 2	MED1086	MED1100	MED1086	Age	Age	MED1086	MED1086	WBC
	MED321	Calcium	Glucose	Temperature	BMI	WBC	Age	MED1086
	MED516	MED582	MED321	Pulse	Pulse	DRG0	WBC	DRG261
	WBC	WBC	WBC	BMI	Systolic BP	COM12	MED321	DRG0
	DRG261	MED746	MED655	WBC	WBC	DRG261	Systolic BP	MED1039
	DRG0	MED321	MED880	Systolic BP	AST	MED516	Pulse	MED677
	Glucose	MED134	MED12	AST	Glucose	Glucose	BMI	MED321
	Bilirubin	Glucose	AST	Diastolic BP	BUN	Temperature	MED516	COM2
	Temperature	MED1086	MED134	MED655	MED655	Bilirubin	Glucose	Calcium
	MED548	Albumin	COM12	MED314	Platelets	COM2	Diastolic BP	DRG3
Stage 3	MED1086	MED321	DRG178	Age	Age	MED1086	Age	Age
	MED321	Glucose	DRG3	Temperature	WBC	DRG0	Systolic BP	MED1086
	DRG0	MED1086	DRG97	Pulse	Pulse	Systolic BP	MED1086	MED321
	Temperature	WBC	MED1086	BMI	BMI	Temperature	Pulse	MED516
	MED516	BMI	COM24	Systolic BP	Systolic BP	WBC	Diastolic BP	Temperature
	DRG3	MED139	CCS71	WBC	AST	MED314	BMI	DRG261
	Systolic BP	MED308	MED314	AST	BUN	DRG3	MED321	DRG0
	DRG261	Systolic BP	BMI	Diastolic BP	Diastolic BP	COM12	Temperature	WBC
	WBC	MED582	DRG0	MED655	Platelets	MED321	WBC	CCS219
	DRG263	MED880	COM12	MED314	Temperature	DRG261	MED314	MED314

Abbreviation: DRG0: Liver transplant; DRG3: Tracheotomy w/dmv w exten proc; DRG97: Maj small & large bowel proc; DRG178: Kidney/urinary trach malignancy; DRG179: Kidney/urinary trach-nonmalig; DRG261: Infect & parasitic disease; DRG263: Septicemia & dissem infect; COM2: Renal failure; COM12: Obesity; COM24: Hypertension; MED12: oxycodone; MED134: benzoic acid; MED139: 1,2,6-hexanetriol; MED308: (all-z)−4,7,10,13,16-docosapentaenoic acid; MED314: lactate; MED319: amphotericin b liposome; MED321: vancomycin; MED516: glucose; MED548: insulin regular, human buffered; MED582: levofloxacin; MED655: calcium chloride; MED677: polyethylene glycol 3350; MED746: insulin, aspart, human/rdna; MED880: heparin, porcine; MED939: amiodarone; MED1039: aldesleukin; MED1086: tazobactam; MED1100: magnesium sulfate; CCS58: Cystic fibrosis; CCS71: Skin and subcutaneous tissue infections; CCS219: Cancer of liver and intrahepatic bile duct.

Traditionally known AKI risk factors include inherent risk factors and medication exposure/disease-related AKI risk factors. Considering the inherent risk factors, namely the susceptibility of each individual patient, age is one of the most important risk factors for AKI, for example, previous research found that age is so substantial that other risk factors (e.g., sepsis, hypertension and nephrotoxins) lost their prediction ability for AKI among patients older than 75 years^17^. In our study, age ranked first by two FS methods as shown in Table 4. Other inherent risk factors for AKI are those associated with reduced kidney reserve or failure of other organs with known cross-talk with the kidneys (e.g., heart, liver, and respiratory system)^18^. As shown in Tables 4 and S4, those related features (e.g. DRG178, DRG179, COM2, COM24, and CCS219) received higher rankings. Medication exposure/disease-related AKI risk factors include exposure to nephrotoxins (i.e., non-steroidal anti-inflammatory drugs, antibiotics), and some disease-related procedures and surgeries, such as vancomycin (MED321), suprofen (MED1212), liver transplant (DRG0), and tracheotomy w/dmv w exten proc (DRG3) were all identified as important factors in this study. Moreover, tazobactam, a known nephrotoxic drug, was ranked second among all risk factors for AKI across four FS methods.

In recent years, there have been several reports regarding novel and previously unknown risk factors for AKI, such as hyperuricemia^19^, hypoalbuminemia^20^, obesity^21^, obstructive sleep apnea^22^, hypochloremia and hyperchloremia^23^. In our study, BMI and obesity (COM2) were both selected in the top ten by several FS methods as shown in Table 4. Furthermore, laxatives medicines such as magnesium sulfate and polyethylene glycol 3350 have been identified by previous studies as prerenal cause of AKI^24^, but were not used in existing AKI prediction models.

Other top-ranked features or potential risk factors warrant further analysis for new knowledge discovery. As shown in Table 4, medication appeared more frequently in the top 10 features across FS methods, which implies their important role in AKI prediction. Since medications are modifiable factors, they can be embedded into clinical decision support systems to generate actionable alerts in effort to prevent AKI.

## Discussion

EMR-based prediction and risk factor discovery is a crucial problem with enormous applications in medicine such as prognosis, patient stratification in clinical trials and prediction of disease risk or response to a given treatment. Feature selection has been extensively studied for many years and has found applications in many domains, especially for problems involving high dimensional data^25–27^. However, stability is a major issue for feature selection, especially in the context of sample variation. Such stability or robustness of the selection process with respect to sample variation has profound impact on the confidence of an expert in the results for further clinical validation. Additionally, from a practical point of view, the ranking list of feature variables from EMR data is often as important as the value of the statistic. Most often the ranking list determines whether the feature would be selected for future analysis in the process of research projects. Hence providing a reliable list of top-ranking features is of great significance.

This study explored the use of FS techniques for AKI prediction and risk factor identification from EMR data. By comparing eight different FS algorithms on a large number of bootstrapped samples, we analyzed and discussed on which FS method is more suitable for AKI prediction from three aspects: stability, similarity between selected features and prediction performance. Some interesting results were found: (1) feature ranking lists showed considerable variability across different data samples and FS techniques; (2) prediction accuracy did not intrinsically guarantee feature stability; (3) prediction performance did not vary significantly across FS methods; however, the final set of selected features was quite different; and (4) a positive correlation was observed between the complexity of suitable FS method and sample size.

An important aspect of FS analysis while under-considered in the literature is the variability of the obtained ordered lists of selected features. Ranked feature lists may be highly instable in the sense that different feature selection method may yield different rankings, and that changes in data set also affects the obtained feature ranking considerably. As shown in Fig. 2, the stability of different FS methods presented diverse trends with the increase of the number of top-k features. In particular, LS (unsupervised multivariate filter method) showed a steady tendency and the stability of ILFS (supervised multivariate filter method) decreases as the number of top-k feature sets while the stability of LLCFS (unsupervised multivariate filter method) increases as top-k increases. Besides, most of filter FS methods are more stable than those complex embedded FS on smaller samples (e.g., AKI-3). With smaller number of samples, the ranking output of univariate Chi-square test outperformed that of most multivariate FS methods in terms of stability.

In short, ideal rankings should have high stability and low bias (i.e., high reliability); however, “reliability” in the context of EMR feature ranking for AKI prediction is difficult to define because the absolute truth is unknown. Therefore, in this study, we chose to assess the reliability not only by prediction performance of AKI using the top-ranked features, but also by comparing the obtained top-ranking features with previous medical knowledge as shown in Supplementary Table S6. Moreover, we observed that the top-ranked features selected by Chi-square method are often those having a higher relative percentage than that of non-AKI samples (e.g., medication factors); the multi-valued discrete variables (such as demographics, patient’s status and lab test categories) would receive higher rankings from the unsupervised multivariate filter methods LS and LLCFS.

### Strengths and Limitations

Our study leveraged nine-years of EMR data containing 76,957 eligible hospital encounters and compared eight widely-applied FS methods for AKI prediction. A positive correlation was observed between the complexity of suitable FS method and sample size. This study provides several practical implications, including recognizing the importance of feature stability as it is desirable for model reproducibility, identifying important AKI risk factors for further investigation, and facilitating early prediction of AKI.

Our analysis has a few limitations. First, the ranking lists were based on a single-center data, and external validation in other institutions would improve generalizability^28^. Second, we limited the analysis to patients who were admitted to the hospital with a minimum eGFR of 60 ml/min/1.73 m^2^ and had normal serum creatinine on the day of admission. Although patients with reduced estimated glomerular filtration rate (eGFR) are at increased risk for AKI, in this study it is difficult to determine which of these patients had hospital-acquired vs community-acquired AKI without adequate longitudinal assessment of kidney function. Third, we only selected lab tests based on previous literature for AKI prediction, not all lab values such as anemia data (i.e., RBC or HGB) were included. Since the selected features varied across AKI stages, identifying specific rules that explain the difference is an interest in our future work. In addition, we also plan to study the issue of adding the important “timing of AKI” and temporal information in EMR for the prediction task. Finally, the study did not use urine output to define AKI nor include it as a risk variable. Although urine output is one of the diagnostic criteria of AKI, it may not be specific enough for designation of AKI because it can be influenced by factors other than renal health and urine output is not frequently collected among the general inpatient population.

In conclusion, our study investigated the behaviors of eight popular state-of-the-art feature selection methods in terms of stability with respect to data sampling variation, similarity between selection results, and AKI prediction performance. Our results illustrated that (1) stability does not intrinsically guarantee prediction accuracy and vice versa, (2) only when the sample size is large enough, complex FS methods should be used, otherwise, a simple FS method is more suitable. Furthermore, many medication features were observed to be important predictors of hospital-acquired AKI, which has important implications for clinical practice and research as they can be embedded into clinical decision support systems to generate actionable alerts for physicians to modify treatment on patients at high AKI risk.

## Methods

### Study Population

A retrospective cohort was built from the University of Kansas Medical Center’s (KUMC) de-identified clinical data repository called HERON (Health Enterprise Repository for Ontological Narration)^29^ containing EMR data from the University of Kansas Health System (KUHS), which is a tertiary academic medical center with >700 staffed beds and >25,000 inpatient admissions per year. No IRB approval was required for this study as the data used met the de-identification criteria specified in the HIPAA Privacy Rule. Our de-identified data request was approved by the HERON Data Request Oversight Committee (DROC) composed of representatives from KUMC and participating clinical organizations.

The research cohort included all adult patients (age at visit ≥18) who were hospitalized for at least two days from November 2007 to December 2016. Given that a patient may have multiple hospital admissions (encounters) of at least two days and develop AKI during one but not another, this study was conducted at the encounter level with a total of 179,370 encounters. From those encounters, we excluded those (a) missing necessary data for outcome determination, i.e. less than two serum creatinine measurements and (b) had evidence of moderate or severe kidney dysfunction at admission, i.e. estimated Glomerular Filtration Rate (eGFR) less than 60 mL/min/1.73 m^2^ or abnormal serum creatinine (SCr) level of >1.3 mg/dL within 24 hours of hospital admission. The final analysis cohort consisted of 76,957 encounters.

### AKI Definition

AKI and its stages of severity were defined according to the Kidney Disease Improving Global Outcomes (KDIGO) serum creatinine criteria^24^ (see Supplementary Table S1). Baseline SCr level was defined as either the last measurement within two-day time window prior to hospital admission or the first SCr measured after hospital admission. All SCr levels measured between admission and discharge were evaluated to determine the occurrence of AKI. Out of the total 76,957 encounters in the final analysis cohort, 7,259 encounters had any AKI of stage 1, 2, or 3 (total 9.43%) and 69,698 had no AKI events.

### Clinical Variables

For each hospital encounter in the final analysis cohort, we extracted EMR data types including demographic information, admission and discharge dates, medications, laboratory values, past medical diagnoses, comorbidities, and admission diagnosis. Details of the clinical variables considered are available in Table 2. This study explored the entirety of the above mentioned EMR data types except for laboratory tests where a selected list of labs that may represent potential presence of a comorbidity correlated with AKI^30^ was considered. SCr and eGFR were not included as predictive variables as they were used to determine the outcome AKI vs non-AKI. Laboratory values were categorized as “unknown”, “less than standard value”, “the standard value”, or “more than the standard value”. Patients’ status was categorized into groups as shown in Supplementary Table S2.

Medication exposure included inpatient (i.e. dispensed during stay) and outpatient medications (i.e. medication reconciliation and prior outpatient prescriptions). All medication names were normalized by mapping to RxNorm ingredient. Comorbidity and admission diagnosis, i.e., all patient refined diagnosis related group (APR-DRG) variables were collected from the University Health System Consortium (UHC; https://www.vizientinc.com) data source in HERON. Patient medical history was captured as major diagnoses (ICD-9 codes grouped according to the Clinical Classifications Software (CCS) diagnosis categories by the Agency for Healthcare Research and Quality). Medical history, medication, comorbidity and admission diagnosis were all binary variables.

### Data Processing

For the patients’ status and laboratory values, variables missing in more than 30% of the population were excluded^31^, otherwise the median value across the entire cohort for the variable was imputed^8^. Only the most recently recorded patients’ status and labs before the AKI prediction point were used for each sample. Medication exposure was defined as true if it is taken within 7-days before the AKI prediction point. Categorical differences were tested with chi-squared tests of homogeneity. Statistical analysis was conducted using MATLAB version R2015b and two-tailed P values < 0.05 denoted statistical significance for all comparisons.

### Feature Selection Methods

Eight popular state-of-the-art feature selection methods were analyzed as representatives of different FS approaches, including: Chi-square test (Chi2), Infinite latent feature selection (ILFS)^32^, Relief-F (ReliefF)^33^, Laplacian score (LS)^34^, Local learning-based clustering with feature selection ILFS (LLCFS)^35^, Minimum redundancy-maximum relevance (mRMR)^36^, Random forests (RF)^37,38^, and Gradient boosting machine (GBM)^39^. Supplementary Table S3 describes their categories and computing complexity. We did not include wrapper methods due to its high computational complexity and inability to produce a ranked list of features.

### Evaluation Protocol

In order to measure stability of feature selection methods with respect to sampling variation, we generated variations of the original dataset through the bootstrapping sampling technique, which is by far one of the most widely used sampling procedures. Since aggregated ranking over multiple subsampled datasets are often believed to be more reliable than rankings obtained from a single dataset, we aggregated a collection of outputs from *t* (here *t* = 100) bootstrap samples by averaging the feature importance scores or coefficients for a specific FS method. Using the aggregated result from each FS method, we assessed prediction performance for each of the AKI stages (1, 2, and 3) vs non-AKI and used similarity index to quantify the variability across multiple FS methods. The entire evaluation protocol is illustrated in Fig. 1.

Let us formalize the evaluation measures as follows. The term ‘data set’ denotes a pair *D = (X, y)*, where the *n* × *m* matrix $X=({x}_{ij}),\,i=1,2,\ldots n;j=1,2,\,\ldots ,\,m.$ If *l* is a ranking list, the $k\,(k\le m)$ top features would be ${l}_{1},\,{l}_{2},\,\ldots ,\,{l}_{k}$. For instance, biomedical articles often report top-20 or top-50 lists. For the sake of simplicity, in this study we considered top-ranking variables only.

#### Stability

Stability over different bootstrapped samples but with the same FS technique was obtained by Kuncheva similarity measure^15^. For a given feature set size *m*, let *t* be the number of bootstrapped datasets, ***s***_*i*_ and ***s***_*j*_ be the selected feature subsets, where $h=|{{\boldsymbol{s}}}_{i}|=|{{\boldsymbol{s}}}_{j}|$, and $r=|{{\boldsymbol{s}}}_{i}{\cap }^{}{{\boldsymbol{s}}}_{j}|$. The Kuncheva Index $=\frac{r\cdot m-{h}^{2}}{h\cdot m-{h}^{2}}$ is a stability index between ***s***_*i*_ and ***s***_*j*_ that takes into account the probability that a feature is selected by chance, which could avoid the tendency to increase when the *h* approaches the *m* and ensures that the stability has high value only if it exceeds the stability by chance^40^. Then stability index can be defined as

1 $$St({{\boldsymbol{s}}}_{i},\,{{\boldsymbol{s}}}_{j})=\frac{2}{t(t-1)}\sum _{i=1}^{t-1}\sum _{j=i+1}^{t}\frac{r\cdot m-{h}^{2}}{h\cdot m-{h}^{2}}\,$$

#### Similarity

We applied Tanimoto distance^41^ to evaluate the similarity of different FS methods. Let ***s***_*i*_, ***s***_*j*_ be the selected feature subsets obtained by *FS*_*i*_ and *FS*_*j*_, respectively. The similarity index is denoted as follows:

2 $$Sim({{\boldsymbol{s}}}_{i},\,{{\boldsymbol{s}}}_{j})=1-\frac{|{{\boldsymbol{s}}}_{i}|+|{{\boldsymbol{s}}}_{j}|-2|{{\boldsymbol{s}}}_{i}{\cap }^{}{{\boldsymbol{s}}}_{j}|}{|{{\boldsymbol{s}}}_{i}|+|{{\boldsymbol{s}}}_{j}|-|{{\boldsymbol{s}}}_{i}{\cap }^{}{{\boldsymbol{s}}}_{j}|}$$

#### Prediction

To compare prediction performance of different FS methods, we implemented Random Forest classifiers^39^ trained over each AKI stage vs non-AKI using the top-k feature ranking set. Random Forest was chosen as the base classifier because it is easy to tune, robust to overfitting, and often demonstrates better performances than other standard classifiers^42^. Area under the receiver operating characteristic (AUC)^43^ curve was calculated as the evaluation metric for prediction performance using a 10-fold cross-validation scheme.

## Electronic supplementary material

Supplementary Information

## Data Availability

Due to patient privacy concern, we will not be able to make the EMR dataset used in this study available to the public. Other materials including methods and programming codes will be made available to all readers.
