# Supplementary material for: Feature Ranking in Predictive Models for Hospital-Acquired Acute Kidney Injury
Source: Sci Rep. 2018 Nov 23;8:17298. doi: 10.1038/s41598-018-35487-0 (PMC6251919; doi:10.1038/s41598-018-35487-0)
Supplement: Supplementary file 1 — Supplementary Information [file 41598_2018_35487_MOESM1_ESM.pdf]

Feature Ranking in Predictive Models for Hospital-Acquired Acute Kidney Injury

Lijuan Wu[1,2†], Yong Hu[1,2†*], Xiaoxiao Liu[1,2†], Xiangzhou Zhang[1,2], Weiqi Chen[1,2], Alan S.L. Yu[3], John A. Kellum[4], Lemuel R. Waitman[5], Mei Liu[5*]

[1]Big Data Decision Institute (BDDI), Jinan University, Guangzhou, 510632, China.
[2]Guangdong Engineering Technology Research Center for Big Data Precision Healthcare, Guangzhou, 510632, China.
[3]Division of Nephrology and Hypertension and the Kidney Institute, University of Kansas Medical Center, Kansas City, 66160, USA.
[4]Center for Critical Care Nephrology, Department of Critical Care Medicine, University of Pittsburgh School of Medicine, Pittsburgh, 15260, USA.
[5]Department of Internal Medicine, Division of Medical Informatics, University of Kansas Medical Center, Kansas City, 66160 USA.

Supplementary Information

Table S1. The KDIGO staging system for AKI

Table S2. Discretization for patients' status

Table S3. Feature selection approaches in the experiments

Table S4. Frequency of top 10 features selected by 8 feature ranking methods

Table S5. Top 50 features selected by 8 feature ranking methods for AKI stages 1-3

Table S6. Summary of the predictors with literature support.

Table S1. The KDIGO staging system for AKI

AKI Stage	Serum Creatinine (SCr) Criteria
1	Increase >26.4 μmol/L (0.3 mg/dL) within 48 hours or 1.5-1.9 times baseline within 7 days
2	Increase 2.0-2.9 times baseline
3	Increase creatinine >354 μmol/L (4.0 mg/dL) or 3 times baseline

Table S2. Discretization for patients' status

Patients' status	Categories
**BMI**	<18.5, [18.5-24.9], [25.0–29.9], >30.0, Unknown
**Diastolic BP**	<80, [80–89], [90–99], >100, Unknown
**Systolic BP**	<120, [120–139], [140–159], >160, Unknown
**Pulse**	<50, [50–65], [66–80], [81–100], >100, Unknown
**Temperature**	<95.0, [95.0–97.6], [97.7–99.5], [99.5–104.0], >104.0, Unknown

Table S3. Feature selection approaches in the experiments

Feature Selection	Uni/multivariate	Type	Class	Computing Complexity
Chi2	Univariate	Filter	Supervised	nm
ILFS	Multivariate	Filter	Supervised	$O(m^{2.37}+im+n+C)$
ReliefF	Multivariate	Filter	Supervised	O($inmC$)
LS	Multivariate	Filter	Unsupervised	N/A
LLCFS	Multivariate	Filter	Unsupervised	N/A
mRMR	Multivariate	Filter	Supervised	$O(m^3n^2)$
RF	Multivariate	Embedded	Supervised	$O(inmlogn)$
GBM	Multivariate	Embedded	Supervised	N/A

As for the complexity, n is the number of samples, m is the number of features, i is the number of iterations, and C is the number of classes. 'N/A' means that it is not documented in the literature. Note that LS can be performed in either supervised or unsupervised fashion, but in this article, we applied the unsupervised class.

Table S4. Frequency of top 10 features selected by 8 feature ranking methods

ID	Name	AKI-1 (N=6,396) n (%)	AKI-2 (N=678) n (%)	AKI-3 (N=185) n (%)	non-AKI (N=69,698) n (%)
Age	Age				
	18-25	303(4.74)	29(4.28)	25(13.51)	4596(6.59)
	26-35	514(8.04)	44(6.49)	23(12.43)	7339(10.53)
	36-45	711(11.12)	76(11.21)	25(13.51)	8601(12.34)
	46-55	1218(19.04)	157(23.17)	35(18.92)	14374(20.62)
	56-64	1672(26.14)	185(27.29)	49(26.49)	16192(23.24)
	>64	1978(30.92)	187(27.56)	28(15.14)	18596(26.68)
BMI	BMI (kg/m^2)				
	Unknown	236(3.69)	12(1.77)	6(3.24)	4844(6.95)
	<18.5	229(3.58)	16(2.36)	5(2.70)	2258(3.24)
	18.5-24.9	1538(24.05)	124(18.29)	36(19.46)	18379(26.37)
	25.0-29.9	1738(27.17)	188(27.73)	36(19.46)	18875(27.08)
	>30.0	2655(41.51)	338(49.85)	102(55.14)	25342(36.36)
Diastolic	Diastolic BP (mmHg)				
	Unknown	9(0.14)	0(0)	0(0)	70(0.10)
	<80	4811(75.22)	496(73.16)	120(64.86)	49768(71.41)
	80-89	1108(17.32)	113(16.67)	32(17.30)	13808(19.81)
	90-99	383(5.99)	57(8.41)	28(15.14)	5035(7.22)
	>100	85(1.33)	12(1.77)	5(2.70)	1017(1.46)
Systolic	Systolic BP				
	Unknown	9(0.14)	0(0)	0(0)	70(0.10)
	<120	2543(39.76)	229(33.78)	44(23.78)	26817(38.48)
	120-139	2412(37.71)	272(40.12)	73(39.46)	28106(40.32)
	140-159	1208(18.89)	142(20.94)	56(30.27)	12671(18.18)
	>160	224(3.50)	35(5.16)	12(6.49)	2034(2.92)
Pulse	Pulse (beats/min)				
	Unknown	9(0.14)	0(0.00)	0(0.00)	69(0.10)
	<50	856(13.38)	66(9.73)	20(10.81)	11206(16.08)
	50-65	2201(34.41)	236(34.81)	65(35.14)	25905(37.17)
	66-80	2451(38.32)	276(40.71)	69(37.30)	25471(36.54)
	81-100	66(1.03)	10(1.47)	4(2.16)	346(0.50)
	>100	813(12.71)	90(13.27)	27(14.59)	6701(9.61)
Temp	Temperature (^{o}F)				
	Unknown	13(0.20)	0(0.00)	0(0.00)	115(0.16)
	<95.0	30(0.47)	4(0.59)	2(1.08)	67(0.10)
	95.0-97.6	4949(77.38)	525(77.43)	138(74.59)	56240(80.69)
	97.7-99.5	195(3.05)	46(6.78)	11(5.95)	1216(1.74)
	99.5-104.0	1(0.02)	0(0.00)	1(0.54)	7(0.01)

	>104.0	1208(18.89)	103(15.19)	33(17.84)	12053(17.29)
Lab0	Albumin (g/dL)				
	Unknown	647(10.12)	71(10.47)	19(10.27)	11652(16.72)
	<3.5	3634(56.82)	405(59.73)	104(56.22)	31367(45.39)
	3.5-5.5	2115(33.06)	202(29.79)	62(33.51)	26398(37.87)
	>5.5	0(0.00)	0(0.00)	0(0.00)	13(0.02)
Lab2	AST (U/L)				
	Unknown	645(10.08)	73(10.77)	20(10.81)	11642(16.70)
	<10	91(1.42)	10(1.47)	2(1.08)	1005(1.44)
	10-40	4271(66.78)	418(61.65)	126(68.11)	46839(67.21)
	>40	1389(21.72)	177(26.11)	37(20.00)	10205(14.64)
Lab4	Calcium (mg/dL)				
	Unknown	2(0.03)	1(0.15)	0(0)	17(0.02)
	<8.5	2615(40.88)	303(44.69)	65(35.14)	22721(32.60)
	8.5-10.2	3705(57.93)	373(55.01)	119(64.32)	46537(66.77)
	>10.2	74(1.16)	1(0.15)	1(0.54)	419(0.60)
Lab5	BUN (mmol/L)				
	Unknown	1(0.02)	1(0.15)	0(0)	16(0.02)
	<7	635(9.93)	81(11.95)	40(21.62)	11278(16.18)
	7-20	4590(71.76)	486(71.68)	135(72.97)	51837(74.38)
	>20	1170(18.29)	110(16.22)	10(5.41)	6563(9.42)
Lab6	Bilirubin (mg/dL)				
	Unknown	652(10.19)	73(10.77)	20(10.81)	11711(16.80)
	<0.3	226(3.53)	18(2.65)	6(3.24)	2920(4.19)
	0.3-1.9	4909(76.75)	503(74.19)	144(77.84)	52115(74.77)
	>1.9	609(9.52)	84(12.39)	15(8.11)	2952(4.24)
Lab9	Glucose (mg/dL)				
	Unknown	2(0.03)	1(0.15)	0(0)	16(0.02)
	<80	302(4.72)	16(2.36)	4(2.16)	3504(5.03)
	80-160	4997(78.13)	514(75.81)	151(81.62)	59206(84.95)
	>160	1095(17.12)	147(21.68)	30(16.22)	6972(10.00)
Lab11	Platelets ($\times 10^9$/L)				
	Unknown	18(0.28)	1(0.15)	1(0.54)	139(0.20)
	<150	1776(27.77)	181(26.70)	41(22.16)	14834(21.28)
	150-450	4324(67.60)	460(67.85)	132(71.35)	52207(74.90)
	>450	278(4.35)	36(5.31)	11(5.95)	2518(3.61)
Lab13	WBC ($\times 10^9$/L)				
	Unknown	18(0.28)	1(0.15)	1(0.54)	136(0.20)
	<3.5	450(7.04)	41(6.05)	7(3.78)	3773(5.41)
	3.5-10.5	3676(57.47)	339(50.00)	103(55.68)	49373(70.84)
	>10.5	2252(35.21)	297(43.81)	74(40.00)	16416(23.55)
DRG0	Liver transplant	126(1.97)	24(3.54)	7(3.78)	147(0.21)
DRG3	Tracheotomy w/dmv w exten proc	99(1.55)	10(1.47)	3(1.62)	80(0.11)
DRG97	Maj small & large bowel proc	147(2.30)	19(2.80)	2(1.08)	423(0.61)
DRG178	Kidney/urinary trach malignancy	59(0.92)	1(0.15)	0(0.00)	264(0.38)

DRG179	Kidney/urinary trach nonmalignancy	92(1.44)	5(0.74)	0(0.00)	416(0.60)
DRG261	Infect & parasitic disease	110(1.72)	31(4.57)	6(3.24)	322(0.46)
DRG263	Septicemia & dissem infect	359(5.61)	46(6.78)	20(10.81)	2420(3.47)
COM2	Renal failure	354(5.53)	43(6.34)	6(3.24)	1377(1.98)
COM12	Obesity	1044(16.32)	75(11.06)	20(10.81)	4379(6.28)
COM24	Hypertension	3373(52.74)	392(57.82)	95(51.35)	31934(45.82)
MED12	Oxycodone	3690(57.69)	411(60.62)	117(63.24)	42493(60.97)
MED134	Benzoic acid	2754(43.06)	265(39.09)	56(30.27)	17751(25.47)
MED139	1,2,6-hexanetriol	4202(65.70)	439(64.75)	125(67.57)	39166(56.19)
MED308	(all-z)-4,7,10,13,16-docosapentaenoic acid	3098(48.44)	324(47.79)	90(48.65)	28898(41.46)
MED314	Lactate	2259(35.32)	297(43.81)	93(50.27)	28307(40.61)
MED319	Amphotericin b liposome	82(1.28)	7(1.03)	1(0.54)	104(0.15)
MED321	Vancomycin	2005(31.35)	301(44.40)	97(52.43)	14266(20.47)
MED516	Glucose	4119(64.40)	510(75.22)	144(77.84)	34019(48.81)
MED548	Insulin regular, human buffered	1173(18.34)	131(19.32)	21(11.35)	6389(9.17)
MED582	Levofloxacin	2098(32.80)	236(34.81)	67(36.22)	16760(24.05)
MED655	Calcium chloride	2505(39.17)	327(48.23)	98(52.97)	29602(42.47)
MED677	Polyethylene glycol 3350	2042(31.93)	183(26.99)	49(26.49)	23349(33.50)
MED746	Insulin, aspart, human/rdna	2621(40.98)	287(42.33)	59(31.89)	20192(28.97)
MED880	Heparin, porcine	2817(44.04)	290(42.77)	71(38.38)	23563(33.81)
MED939	Amiodarone	744(11.63)	74(10.91)	10(5.41)	3512(5.04)
MED1039	Aldesleukin	142(2.22)	12(1.77)	0(0.00)	107(0.15)
MED1086	Tazobactam	1899(29.69)	285(42.04)	92(49.73)	11385(16.33)
MED1100	Magnesium sulfate	3082(48.19)	336(49.56)	61(32.97)	26682(38.28)
CCS58	Cystic fibrosis	211(3.30)	8(1.18)	4(2.16)	979(1.40)
CCS71	Skin and subcutaneous tissue infections	482(7.54)	60(8.85)	25(13.51)	4219(6.05)
CCS219	Cancer of liver and intrahepatic bile duct	107(1.67)	21(3.10)	7(3.78)	641(0.92)

Table S5. Top 50 features selected by 8 feature ranking methods for AKI stages 1-3

AKI	Chi2	ILFS	ReliefF	LS	LLCFS	mRMR	RF	GBM
**Stage 1**	MED134	MED746	MED1086	Age	Pulse	MED134	Age	WBC
Top-2	MED1086	MED1100	WBC	Temperature	Systolic BP	MED1086	Pulse	MED1086
Top-3	MED516	MED582	MED321	Pulse	BMI	WBC	WBC	MED1039
Top-4	BUN	MED880	Glucose	WBC	WBC	Glucose	BMI	MED134
Top-5	WBC	MED308	MED12	BMI	BUN	MED548	Systolic BP	CCS58
Top-6	MED548	Calcium	MED880	Systolic BP	Age	MED1039	MED1086	BUN
Top-7	MED746	Glucose	MED134	AST	Glucose	BUN	MED134	COM24
Top-8	MED939	MED134	Calcium	BUN	AST	DRG0	BUN	DRG179
Top-9	MED321	WBC	Age	Glucose	Bilirubin	DRG3	Calcium	DRG97
Top-10	MED880	MED139	MED677	Diastolic BP	Temperature	COM2	MED516	MED319
Top-11	MED149	COM14	MED139	ALT	Diastolic BP	MED516	Diastolic BP	Pulse
Top-12	Glucose	Albumin	MED1009	MED655	Platelets	Bilirubin	Glucose	MED321
Top-13	MED310	AST	MED464	MED314	Calcium	CCS230	MED321	Calcium
Top-14	Bilirubin	COM16	MED1078	COM24	MED308	MED464	Platelets	BMI
Top-15	CCS118	COM27	MED548	MED1078	MED12	MED319	COM24	Bilirubin
Top-16	MED1152	MED236	DRG259	MED746	MED1133	DRG261	MED1133	DRG3
Top-17	AST	MED762	BMI	CCS199	ALT	MED880	Gender	DRG178
Top-18	MED582	MED321	MED41	MED134	MED1078	COM10	MED880	Glucose
Top-19	COM10	MED41	MED746	MED41	MED1100	COM0	Albumin	COM2
Top-20	COM2	Glucose	MED689	MED1100	MED880	DRG68	MED12	Age
Top-21	MED139	MED1086	Albumin	MED66	MED677	Temperature	MED746	DRG2
Top-22	MED1039	COM24	COM7	MED437	Gender	Calcium	MED677	MED566
Top-23	Calcium	CCS55	COM0	Bilirubin	MED134	COM13	MED464	MED464
Top-24	MED1100	MED1212	MED888	MED566	MED746	MED1212	MED139	MED516
Top-25	MED464	ALT	MED582	MED308	CCS161	MED746	Temperature	CCS230
Top-26	MED873	MED12	MED155	MED139	MED41	MED96	MED236	MED1111
Top-27	CCS230	MED1191	MED249	MED516	COM24	DRG69	Bilirubin	MED853
Top-28	CCS247	Pulse	MED334	MED147	MED516	AST	MED308	AST
Top-29	COM28	CCS52	CCS161	MED880	MED582	MED321	MED548	MED880
Top-30	DRG0	MED853	MED717	MED12	MED1191	MED572	AST	DRG0
Top-31	MED1000	MED1111	MED1133	Platelets	MED437	DRG49	MED1100	DRG90
Top-32	MED853	MED630	AST	CCS61	MED66	MED939	MED1078	MED334
Top-33	MED79	CCS247	Glucose	MED464	MED655	COM11	MED334	COM16
Top-34	DRG3	CCS199	Gender	MED608	MED1086	DRG90	MED41	MED12
Top-35	MED566	MED464	MED66	MED1133	COM14	COM22	MED1191	DRG261
Top-36	MED155	MED836	BUN	CCS161	MED314	Pulse	MED1111	COM23
Top-37	MED249	MED1048	MED1100	MED836	MED321	MED937	CCS161	MED1212
Top-38	MED147	MED787	MED55	MED582	CCS199	MED853	MED582	COM13
Top-39	DRG68	MED821	MED174	Calcium	Albumin	COM27	MED1212	DRG128
Top-40	COM11	MED187	Pulse	MED1152	MED1075	COM16	MED437	MED79
Top-41	DRG69	Diastolic BP	MED939	CCS247	MED762	DRG66	Race1	DRG69
Top-42	Platelets	MED334	MED1191	MED1191	MED236	MED659	CCS199	MED41
Top-43	Age	DRG131	MED594	CCS127	MED334	DRG140	COM14	Temperature

Top-44	MED1231	MED1009	MED187	COM28	MED464	COM23	MED1075	MED677
Top-45	MED836	CCS124	MED1111	MED98	MED138	COM12	COM0	DRG68
Top-46	COM13	DRG259	MED308	Albumin	MED608	DRG128	COM16	DRG84
Top-47	MED762	CCS230	CCS52	MED787	Race1	CCS209	MED853	CCS118
Top-48	Pulse	Bilirubin	MED853	MED321	MED594	DRG284	MED66	MED187
Top-49	COM14	MED436	Bilirubin	MED677	CCS55	MED1008	MED138	COM10
Top-50	CCS15	MED943	MED268	MED334	CCS52	CCS118	MED1230	MED548
**Stage 2**	MED1086	MED1100	MED1086	Age	Age	MED1086	MED1086	WBC
Top-2	MED321	Calcium	Glucose	Temperature	BMI	WBC	Age	MED1086
Top-3	MED516	MED582	MED321	Pulse	Pulse	DRG0	WBC	DRG261
Top-4	WBC	WBC	WBC	BMI	Systolic BP	COM12	MED321	DRG0
Top-5	DRG261	MED746	MED655	WBC	WBC	DRG261	Systolic BP	MED1039
Top-6	DRG0	MED321	MED880	Systolic BP	AST	MED516	Pulse	MED677
Top-7	Glucose	MED134	MED12	AST	Glucose	Glucose	BMI	MED321
Top-8	Bilirubin	Glucose	AST	Diastolic BP	BUN	Temperature	MED516	COM2
Top-9	Temperature	MED1086	MED134	MED655	MED655	Bilirubin	Glucose	Calcium
Top-10	MED548	Albumin	COM12	MED314	Platelets	COM2	Diastolic BP	DRG3
Top-11	MED149	MED689	BMI	Glucose	Gender	DRG3	BUN	BMI
Top-12	MED1152	MED880	MED334	COM24	Temperature	COM22	Temperature	Pulse
Top-13	AST	COM24	Albumin	ALT	MED1133	MED321	Calcium	Diastolic BP
Top-14	COM12	Glucose	Calcium	BUN	MED677	MED548	DRG261	Glucose
Top-15	DRG3	COM14	MED677	MED566	Albumin	MED96	AST	MED516
Top-16	MED1008	BMI	COM24	CCS199	MED308	Calcium	COM12	MED864
Top-17	MED134	AST	MED1078	MED1078	MED1100	COM10	Gender	Temperature
Top-18	MED746	MED1009	MED582	MED41	Calcium	MED1008	Bilirubin	Age
Top-19	MED281	COM16	MED134	MED147	ALT	MED864	Platelets	MED746
Top-20	MED310	MED762	Glucose	MED746	MED147	MED281	MED1133	MED566
Top-21	COM3	Pulse	COM12	MED437	CCS161	COM16	COM24	AST
Top-22	COM2	MED655	MED41	MED1100	MED746	COM24	MED677	COM21
Top-23	MED939	CCS199	MED1230	MED139	CCS199	AST	MED12	COM12
Top-24	MED96	Race3	MED1075	MED516	MED134	MED1039	COM16	COM16
Top-25	COM10	MED1111	MED821	MED464	MED1075	DRG66	DRG0	MED319
Top-26	BMI	MED1191	Race4	MED308	MED762	Pulse	MED1191	MED501
Top-27	COM22	MED1152	Bilirubin	MED134	MED66	DRG262	MED1100	MED1008
Top-28	DRG66	DRG259	MED1152	MED12	MED314	MED1152	COM14	COM24
Top-29	Calcium	MED1078	MED746	MED66	MED608	DRG115	MED139	CCS55
Top-30	MED864	CCS225	MED464	MED880	MED41	COM3	MED746	MED1133
Top-31	MED1039	MED677	Systolic BP	CCS61	Diastolic BP	MED319	MED236	MED314
Top-32	Pulse	MED138	COM3	Platelets	MED566	COM21	Albumin	MED362
Top-33	COM16	MED314	COM7	MED1133	COM24	MED133	Race1	DRG49
Top-34	MED582	MED334	CCS61	CCS161	MED836	MED497	MED1078	MED464
Top-35	COM24	MED890	MED1111	MED98	MED321	DRG309	MED334	DRG262
Top-36	BUN	CCS58	MED139	MED1191	Bilirubin	MED572	MED308	DRG309
Top-37	CCS182	MED1123	MED866	Bilirubin	MED236	DRG49	MED464	DRG97
Top-38	MED133	DRG263	MED6	MED608	MED880	MED1076	CCS199	COM14

Top-39	MED762	DRG297	BUN	MED582	MED787	COM14	MED41	MED281
Top-40	COM14	COM27	CCS127	Albumin	MED582	MED501	MED134	MED417
Top-41	MED79	CCS62	COM16	Gender	MED803	MED362	MED880	COM22
Top-42	DRG262	MED974	MED236	Calcium	MED516	DRG30	MED582	Bilirubin
Top-43	COM11	MED139	Race1	CCS247	MED12	CCS219	MED314	Race2
Top-44	MED1100	MED698	CCS225	MED677	MED1078	CCS182	MED1212	DRG66
Top-45	CCS219	MED1044	COM0	MED888	MED1152	COM23	MED1111	MED1152
Top-46	CCS225	CCS168	MED594	MED174	MED1111	DRG7	MED1152	MED833
Top-47	MED873	MED464	MED203	COM28	CCS79	BUN	CCS161	Race1
Top-48	MED880	MED630	CCS199	MED836	MED98	DRG119	MED281	MED497
Top-49	COM21	CCS125	COM14	Race1	CCS175	MED417	MED655	COM23
Top-50	MED319	CCS53	CCS182	CCS127	MED464	DRG116	MED1075	MED635
**Stage 3**	MED1086	MED321	DRG178	Age	Age	MED1086	Age	Age
Top-2	MED321	Glucose	DRG3	Temperature	WBC	DRG0	Systolic BP	MED1086
Top-3	DRG0	MED1086	DRG97	Pulse	Pulse	Systolic BP	MED1086	MED321
Top-4	Temperature	WBC	MED1086	BMI	BMI	Temperature	Pulse	MED516
Top-5	MED516	BMI	COM24	Systolic BP	Systolic BP	WBC	Diastolic BP	Temperature
Top-6	DRG3	MED139	CCS71	WBC	AST	MED314	BMI	DRG261
Top-7	Systolic BP	MED308	MED314	AST	BUN	DRG3	MED321	DRG0
Top-8	DRG261	Systolic BP	BMI	Diastolic BP	Diastolic BP	COM12	Temperature	WBC
Top-9	WBC	MED582	DRG0	MED655	Platelets	MED321	WBC	CCS219
Top-10	DRG263	MED880	COM12	MED314	Temperature	DRG261	MED314	MED314
Top-11	BMI	COM24	MED321	COM24	MED1100	DRG49	MED41	MED102
Top-12	Age	MED314	MED516	ALT	Gender	MED497	MED12	DRG4
Top-13	Diastolic BP	MED1191	Systolic BP	Glucose	MED1133	MED686	Albumin	Diastolic BP
Top-14	COM12	MED236	BMI	BUN	MED655	CCS219	BUN	Systolic BP
Top-15	CCS71	MED655	Diastolic BP	MED566	Glucose	MED140	MED1100	DRG262
Top-16	DRG262	MED1241	MED12	CCS199	MED677	CCS175	Calcium	MED746
Top-17	Pulse	MED677	MED582	MED1078	MED12	DRG4	Platelets	DRG30
Top-18	DRG49	CCS225	MED1100	MED147	Albumin	DRG119	Race1	DRG49
Top-19	MED140	CCS23	MED1191	MED41	MED308	CCS135	COM24	DRG219
Top-20	CCS219	MED937	COM16	MED437	ALT	MED516	MED1078	MED408
Top-21	DRG4	MED1159	WBC	MED746	MED516	DRG115	MED1191	DRG2
Top-22	MED582	MED1078	MED655	MED464	Calcium	COM17	MED308	AST
Top-23	CCS260	CCS77	COM24	MED1100	MED880	DRG262	Gender	DRG263
Top-24	Glucose	CCS116	MED139	MED139	MED314	Glucose	MED334	BMI
Top-25	CCS182	CCS219	MED310	MED516	MED236	DRG186	MED677	MED1027
Top-26	MED532	COM7	Glucose	MED12	MED1075	MED1060	MED516	Bilirubin
Top-27	MED876	CCS133	MED630	MED308	CCS161	MED128	COM16	Glucose
Top-28	MED497	MED1244	MED41	MED66	MED147	DRG153	MED880	MED677
Top-29	MED664	CCS109	AST	MED134	COM24	CCS71	Race2	MED516
Top-30	MED139	COM13	MED880	CCS61	MED41	COM1	MED1212	MED352
Top-31	MED175	CCS16	MED953	MED880	CCS199	CCS30	MED139	MED41
Top-32	MED1152	MED334	CCS165	Platelets	MED66	DRG263	Glucose	MED686
Top-33	MED155	CCS67	COM12	MED1133	MED566	MED1142	MED437	MED1133

Top-34	MED249	Platelets	CCS225	MED98	MED582	MED1119	COM12	MED6
Top-35	MED310	CCS223	BUN	CCS161	MED464	MED256	MED236	MED140
Top-36	MED686	MED664	Albumin	MED1191	MED134	Pulse	DRG263	MED1212
Top-37	COM11	CCS22	Bilirubin	Gender	MED746	CCS260	MED134	MED1017
Top-38	MED256	COM1	MED1048	Albumin	MED1212	DRG25	MED582	DRG3
Top-39	COM17	Race3	CCS232	Calcium	MED1078	MED584	COM14	MED600
Top-40	Bilirubin	COM12	MED1009	MED608	MED762	MED692	MED464	MED334
Top-41	MED655	Diastolic BP	MED260	MED582	Bilirubin	DRG179	MED746	MED464
Top-42	DRG2	CCS211	MED348	MED888	MED321	MED876	CCS161	COM2
Top-43	BUN	MED1040	MED1172	MED677	MED608	DRG116	COM27	DRG68
Top-44	MED73	CCS130	COM28	CCS247	MED139	DRG113	COM0	MED73
Top-45	CCS175	MED866	COM3	MED174	Race1	MED589	MED888	MED138
Top-46	MED314	MED813	MED436	Race1	MED1111	MED102	MED655	MED510
Top-47	MED244	MED689	CCS265	MED236	MED98	COM11	MED314	MED873
Top-48	MED1119	COM17	CCS116	COM28	MED787	DRG111	MED510	MED572
Top-49	ALT	CCS146	CCS247	Bilirubin	MED437	MED154	Bilirubin	MED1100
Top-50	Race2	MED66	MED689	MED836	COM14	DRG13	CCS199	DRG140

Abbreviation: Race1: White; Race2: African American; Race3: Asian; Race4: Other Race; COM0: Diabetes w/CCs; COM1: Hypothyroidism; COM2: Renal failure; COM3: Liver disease; COM7: Metastatic cancer; COM10: Congestive Heart Failure; COM11: Coagulopthy; COM12: Obesity; COM13: Weight loss; COM14: Fluid and electr disorders; COM16: Deficiency Anemias; COM17: Alcohol abuse; COM21: Valvular disease; COM22: Pulmonary circulation disease; COM23: Peripheral vascular disease; COM24: Hypertension; COM27: Chronic pulmonary disease; COM28: Diabetes w/o CCs; DRG0: Liver transplant; DRG2: Bone marrow transplant; DRG3: Trach w/dmv w exten proc; DRG4: Trach w/dmv w/o enten proc; DRG7: Craniotomy exc for trauma; DRG13: Nervous system neoplasms; DRG25: Seizure; DRG30: Oth disorders nervous sys; DRG49: Respiratory system diag; DRG66: Cardiac valve proc w/cath; DRG68: Coronary bypass w/card cath; DRG69: Cor bypass w/o card cath; DRG84: Heart failure; DRG90: Card arrhythmia & conduction; DRG97: Maj small & large bowel proc; DRG111: Inflammatory bowel disease; DRG113: Intestinal obstruction; DRG115: Non-bacterial gi; DRG116: Abdominal pain; DRG119: Oth digestive system diag; DRG128: Pancreas disord exc malig; DRG131: Hip joint replacement; DRG140: Knee & lower leg proc; DRG153: Back & neck disorders; DRG178: Kidney/urinary tract malig; DRG179: Kidney/urin tract-nonmalig; DRG186: Kidney/urin tract infect; DRG219: Other antepartum diagnoses; DRG259: Chemotherapy; DRG261: Infect & parasitic disease; DRG262: Post-op/trauma infect proc; DRG263: Septicemia & dissem infect; DRG284: Alcohol abuse & dependence; DRG297: Rehabilitation; DRG309: Extensive proc unrel pdx; MED6: desflurane; MED12: oxycodone; MED41: bisacodyl; MED55: 4-((4,6-bis(octylthio)-1,3,5-triazin-2-yl)amino)-2,6-bis(1,1-dimethylethyl)-phenol; MED66: aluminium hydroxide; MED73: voriconazole; MED79: prochlorperazine; MED96: bicyclo; MED98: sevoflurane; MED128: clopidogrel bisulfate; MED133: nitroprusside; MED134: benzoic acid; MED138: methylene blue; MED139: 1,2,6-hexanetriol; MED140: hypochlorite; MED147: 3-((l-menthyl)oxy)propane-1,2-diol; MED149: albumin human; MED154: idarubicin; MED155: gly; MED174: potassium chloride; MED175: propiolactone; MED187: trisulfapyrimidines; MED203: metformin; MED236: hydrocodone; MED244: mannitol; MED249: argipressin; MED256: fludarabine; MED260: atorvastatin; MED268: fluticasone; MED281: mercaptopurine; MED308: (all-z)-4,7,10,13,16-docosapentaenoic acid,; MED310: aminocaproic acid; MED314: lactate; MED319: amphotericin b liposome; MED321: vancomycin; MED334: paracetamol; MED348: nystatin; MED352: caspofungin; MED362: acetylcysteine (obsolete); MED408: calcium gluconate; MED417: methotrexate; MED436: calcium (as carbonate); MED437: 1-(4-(4-chlorophenyl)-3-phenyl-2-butenyl)-pyrrolidine 1,5-naphthalenedisulfonate; MED464: dexamethasone; MED497: cisatracurium; MED501: colistin; MED510: esomeprazole; MED516: glucose; MED532: formaldehyde; MED548: insulin regular,human buffered; MED566: glycopyrronium bromide; MED572: diazepam; MED582: levofloxacin; MED584: naproxen; MED589: bumetanide; MED594: prednisone; MED600: insulin detemir; MED608: simethicone; MED630: lidocaine (obsolete); MED635: olive leaf; MED655: calcium chloride; MED659: ursodeoxycholate; MED664: influenza b virus; MED677: polyethylene glycol 3350; MED686: pentaerithrityl tetranitrate; MED689: famotidine; MED692: pancuronium; MED698: soybean oil; MED717: methylprednisolone; MED746: insulin,aspart,human/rdna; MED762: sodium bicarb (obsolete); MED787:

acetylsalicylic acid; MED803: ethyl chloride; MED813: ether; MED821: sulfonamide; MED833: vecuronium; MED836: glyceryl trinitrate; MED853: carvedilol; MED864: hydroxyethyl starch 130-0.4; MED866: vinylbital; MED873: spironolactone; MED876: micafungin; MED880: heparin,porcine; MED888: bacitracin; MED890: dibasic sod ph (obsolete); MED937: ropivacaine; MED939: amiodarone; MED943: insulin, isophane; MED953: codeine; MED974: sodium phosphate,monobasic; MED1000: alteplase; MED1008: vasopressin; MED1009: diphenhydramine; MED1017: valine; MED102: amphotericin b; MED1027: deslanoside; MED1039: aldesleukin; MED1040: sodium gluconate; MED1044: phospholipids,egg; MED1048: ferrous sulfate; MED1060: indole; MED1075: imidazole; MED1076: coagulation factor viia; MED1078: magnesium hydroxide; MED1086: tazobactam; MED1100: magnesium sulfate; MED1111: aminobutyrate; MED1119: basiliximab; MED1123: cyclobenzaprine; MED1133: benzimidazole; MED1142: acetazolamide; MED1152: albumin; MED1159: potassium; MED1172: bupivacaine; MED1191: docusate; MED1212: suprofen; MED1230: acetaminophen; MED1231: atropine; MED1241: zinc sulfate; MED1244: valproate; CCS15: Respiratory failure; insufficiency; arrest (adult); CCS16: Pleurisy; pneumothorax; pulmonary collapse; CCS22: Esophageal disorders; CCS23: Conditions associated with dizziness or vertigo; CCS30: Melanomas of skin; CCS52: Deficiency and other anemia; CCS53: Other nutritional; endocrine; and metabolic disorders; CCS55: Fluid and electrolyte disorders; CCS58: Cystic fibrosis; CCS61: Disorders of lipid metabolism; CCS62: Nutritional deficiencies; CCS67: Chronic ulcer of skin; CCS71: Skin and subcutaneous tissue infections; CCS77: Aortic; peripheral; and visceral artery aneurysms; CCS79: Other circulatory disease; CCS109: E Codes: Motor vehicle traffic (MVT); CCS116: Syncope; CCS118: Congestive heart failure; nonhypertensive; CCS124: Nonspecific chest pain; CCS125: Pulmonary heart disease; CCS127: Coronary atherosclerosis and other heart disease; CCS130: Other and ill-defined heart disease; CCS133: Leukemias; CCS135: Cancer of kidney and renal pelvis; CCS146: E Codes: Adverse effects of medical drugs; CCS161: Residual codes; unclassified; CCS165: Allergic reactions; CCS168: Medical examination/evaluation; CCS175: Cardiac dysrhythmias; CCS182: Septicemia (except in labor); CCS199: Essential hypertension; CCS209: Other diseases of kidney and ureters; CCS211: Heart valve disorders; CCS219: Cancer of liver and intrahepatic bile duct; CCS223: Blindness and vision defects; CCS225: Other liver diseases; CCS230: Acute and unspecified renal failure; CCS232: Urinary tract infections; CCS247: Diabetes mellitus without complication; CCS260: Infective arthritis and osteomyelitis (except that caused by tuberculosis or sexually transmitted disease); CCS265: Other non-traumatic joint disorders.

Table S6. Summary of the predictors with literature support.

Categories	Name	#	Cite
Demographics	age	31	1-31
	gender	17	7,10-12,14-17,19,20,23,27,28,31-34
	race	1	16
Patients' Status	BMI	7	4,14,16,23,26,30,35
	systolic BP	2	11,24
	diastolic BP	1	11
	temperature	2	11,16
Lab Tests	WBC	4	11,16,27,31
	BUN	3	8,11,16
	AST	3	10,11,16
	ALT	2	16,28
	glucose	4	11,16,27,36
	albumin	2	11,16
	calcium	2	11,16
	platelets	4	3,8,11,16
	total bilirubin	3	8,11,16
Diseases	shock	3	13,18,20
	obesity	2	20,21
	weight loss	1	35
	hypertension	18	2-4,9,13,16,18-21,23,26,31,32,37-40
	renal failure	7	2,7,19,21,28,37,41
	liver disease	3	16,30,37
	alcohol abuse	1	16
	CVA w infarct	1	16
	congestive heart failure	8	14,16,20,31,33,37,41,42
	chronic pulmonary disease	3	13,18,26
	septicemia/infection	2	37,43
	septicemia (except in labor)	2	22,27
	peripheral vascular disease	5	16,23,26,31,41
	acute myocardial infarction	2	22,27
	diabetes	19	4,5,13,16-23,28,31,33,38,40,42,44
	anemias	6	9,13,16,18,22,37
	cancer	2	16,21
	chronic obstructive lung disease	5	14,16,30,33,45
	coronary atherosclerosis and other heart disease	1	44
Medications	lactate	3	4,39,46
	vancomycin	1	16
	sodium bicarb (obsolete)	1	16
	acetylcysteine (obsolete)	1	16
	antiFungals	1	16
	beta blockers	1	16
	vasopressor	2	5,38
	lipid-lowering treatment	1	26

NSAIDs	2	[16,27]
diuretics	3	[27,38,47]
antibiotic	1	[27]
glucocorticoids	1	[16]
insulin	2	[16,32]

NOTE: three categories, including comorbidities (COM), admission diagnosis (DRG), and medical history (CCS), were merged into one category (i.e., diseases).

1 Araujo, G. N. *et al.* Simplifying contrast-induced acute kidney injury prediction after primary percutaneous coronary intervention: the age, creatinine and ejection fraction score. *Cardiovascular intervention and therapeutics* **33**, 224-231 (2018).

2 Zambetti, B. R. *et al.* A web-based tool to predict acute kidney injury in patients with ST-elevation myocardial infarction: Development, internal validation and comparison. *PloS one* **12**, e0181658 (2017).

3 Wang, L. *et al.* Electronic health record-based predictive models for acute kidney injury screening in pediatric inpatients. *Pediatric research* **82**, 465 (2017).

4 Smith, L. E., Smith, D. K., Blume, J. D., Siew, E. D. & Billings, F. T. Latent variable modeling improves AKI risk factor identification and AKI prediction compared to traditional methods. *BMC nephrology* **18**, 55 (2017).

5 Mao, Y. & Qin, Z.-H. Risk factors of acute kidney injury and dialysis among patients attending intensive care units in China. *Int J Clin Exp Med* **10**, 12056-12067 (2017).

6 Duan, C. *et al.* A new preprocedure risk score for predicting contrast-induced acute kidney injury. *Canadian Journal of Cardiology* **33**, 714-723 (2017).

7 Zhou, L. Z. *et al.* Development and validation of a risk score for prediction of acute kidney injury in patients with acute decompensated heart failure: A prospective cohort study in China. *Journal of the American Heart Association* **5**, e004035 (2016).

8 Sanchez-Pinto, L. N. & Khemani, R. G. Development of a prediction model of early acute kidney injury in critically ill children using electronic health record data. *Pediatric Critical Care Medicine* **17**, 508-515 (2016).

9 Nah, C. W. *et al.* A clinical score to predict acute kidney injury after cardiac surgery in a Southeast-Asian population. *Interactive cardiovascular and thoracic surgery* **23**, 757-761 (2016).

10 Liu, X. *et al.* A predictive model for assessing surgery-related acute kidney injury risk in hypertensive patients: a retrospective cohort study. *PloS one* **11**, e0165280 (2016).

11 Koyner, J. L., Adhikari, R., Edelson, D. P. & Churpek, M. M. Development of a multicenter ward–based AKI prediction model. *Clinical Journal of the American Society of Nephrology*, CJN. 00280116 (2016).

12 Jiang, W. *et al.* Dynamic predictive scores for cardiac surgery–associated acute kidney injury. *Journal of the American Heart Association* **5**, e003754 (2016).

13 Inohara, T. *et al.* Performance and validation of the US NCDR acute kidney injury prediction model in Japan. *Journal of the American College of Cardiology* **67**, 1715-1722 (2016).

14 Kristovic, D. *et al.* Cardiac surgery-associated acute kidney injury: risk factors analysis and comparison of prediction models. *Interactive cardiovascular and thoracic surgery* **21**, 366-373 (2015).

15 Gursoy, M., Hokenek, A. F., Duygu, E., Atay, M. & Yavuz, A. Clinical SYNTAX score can predict acute kidney injury following on-pump but not off-pump coronary artery bypass surgery. *Cardiorenal medicine* **5**, 297-305 (2015).

16 Cronin, R. M. *et al.* National Veterans Health Administration inpatient risk stratification models for hospital-acquired acute kidney injury. *Journal of the American Medical Informatics Association* **22**, 1054-1071 (2015).

17 Bell, S. *et al.* Risk of postoperative acute kidney injury in patients undergoing orthopaedic surgery—development and validation of a risk score and effect of acute kidney injury on survival: observational cohort study. *Bmj* **351**, h5639 (2015).

18 Tsai, T. T. *et al.* Validated contemporary risk model of acute kidney injury in patients undergoing percutaneous coronary interventions: insights from the National Cardiovascular Data Registry Cath‐PCI Registry. *Journal of the American Heart Association* **3**, e001380 (2014).

19 Simonini, M. *et al.* A new clinical multivariable model that predicts postoperative acute kidney injury: impact of endogenous ouabain. *Nephrology Dialysis Transplantation* **29**, 1696-1701 (2014).

20 Ng, S. Y. *et al.* Prediction of acute kidney injury within 30 days of cardiac surgery. *The Journal of thoracic and cardiovascular surgery* **147**, 1875-1883. e1871 (2014).

21 Cruz, D. N. *et al.* Utilization of small changes in serum creatinine with clinical risk factors to assess the risk of AKI in critically lll adults. *Clinical Journal of the American Society of Nephrology* **9**, 663-672 (2014).

22 Chen, Y. L. *et al.* A simple preprocedural score for risk of contrast‐induced acute kidney injury after percutaneous coronary intervention. *Catheterization and Cardiovascular Interventions* **83**, E8-E16 (2014).

23 Birnie, K. *et al.* Predictive models for kidney disease: improving global outcomes (KDIGO) defined acute kidney injury in UK cardiac surgery. *Critical Care* **18**, 606 (2014).

24 Wang, Y. n., Cheng, H., Yue, T. & Chen, Y. p. Derivation and validation of a prediction score for acute kidney injury in patients hospitalized with acute heart failure in a Chinese cohort. *Nephrology* **18**, 489-496 (2013).

25 Kim, W. H. *et al.* Simplified clinical risk score to predict acute kidney injury after aortic surgery. *Journal of cardiothoracic and vascular anesthesia* **27**, 1158-1166 (2013).

26 Berg, K. S., Stenseth, R., Wahba, A., Pleym, H. & Videm, V. How can we best predict acute kidney injury following cardiac surgery?: a prospective observational study. *European Journal of Anaesthesiology (EJA)* **30**, 704-712 (2013).

27 Matheny, M. E. *et al.* Development of inpatient risk stratification models of acute kidney injury for use in electronic health records. *Medical Decision Making* **30**, 639-650 (2010).

28 Slankamenac, K. *et al.* Development and validation of a prediction score for postoperative acute renal failure following liver resection. *Annals of surgery* **250**, 720-728 (2009).

29 Palomba, H., De Castro, I., Neto, A., Lage, S. & Yu, L. Acute kidney injury prediction following elective cardiac surgery: AKICS Score. *Kidney international* **72**, 624-631 (2007).

30 Kheterpal, S. *et al.* Predictors of postoperative acute renal failure after noncardiac surgery in patients with previously normal renal function. *Anesthesiology: The Journal of the American Society of Anesthesiologists* **107**, 892-902 (2007).

31 Brown, J. R. *et al.* Multivariable prediction of renal insufficiency developing after cardiac surgery. *Circulation* **116**, I-139-I-143 (2007).

32 Jain, A., McDonald, H. I., Nitsch, D., Tomlinson, L. & Thomas, S. L. Risk factors for developing acute kidney injury in older people with diabetes and community-acquired pneumonia: a population-based UK cohort study. *BMC nephrology* **18**, 142 (2017).

33 Crosina, J. *et al.* Improving the Prediction of Cardiac Surgery–Associated Acute Kidney Injury. *Kidney international reports* **2**, 172-179 (2017).

34 Ohnuma, T. *et al.* External validation for acute kidney injury severity scores: a multicenter retrospective study in 14 Japanese ICUs. *American journal of nephrology* **42**, 57-64 (2015).

35 Park, M. H. *et al.* Clinical risk scoring models for prediction of acute kidney injury after living donor liver transplantation: a retrospective observational study. *PLoS One* **10**, e0136230 (2015).

36 Park, H. S. *et al.* Contrast volume/raw eGFR ratio for predicting contrast-induced acute kidney injury in patients undergoing percutaneous coronary intervention for myocardial infarction. *Cardiorenal medicine* **5**, 61-68 (2015).

37 Malhotra, R. *et al.* A risk prediction score for acute kidney injury in the intensive care unit. *Nephrology Dialysis Transplantation* **32**, 814-822 (2017).

38 Kane-Gill, S. L. *et al.* Risk factors for acute kidney injury in older adults with critical illness: a retrospective cohort study. *American journal of kidney diseases* **65**, 860-869 (2015).

39 Rueggeberg, A. *et al.* Development of a risk stratification model for predicting acute renal failure in orthotopic liver transplantation recipients. *Anaesthesia* **63**, 1174-1180 (2008).

40 Inohara, T. *et al.* Development and validation of a pre-percutaneous coronary intervention risk model of contrast-induced acute kidney injury with an integer scoring system. *The American journal of cardiology* **115**, 1636-1642 (2015).

41 Hougardy, J.-M. *et al.* Chronic kidney disease as major determinant of the renal risk related to on-pump cardiac surgery: a single-center cohort study. *Acta Chirurgica Belgica* **116**, 217-224 (2016).

42 Pannu, N. *et al.* A new model to predict acute kidney injury requiring renal replacement therapy after cardiac surgery. *Canadian Medical Association Journal*, cmaj. 151447 (2016).

43 Zang, H. *et al.* Incidence, risk factors and outcomes of acute kidney injury (AKI) in patients with acute-on-chronic liver failure (ACLF) of underlying cirrhosis. *Hepatology international* **10**, 807-818 (2016).

44 Kate, R. J., Perez, R. M., Mazumdar, D., Pasupathy, K. S. & Nilakantan, V. Prediction and detection models for acute kidney injury in hospitalized older adults. *BMC medical informatics and decision making* **16**, 39 (2016).

45 Eriksen, B. O., Hoff, K. R. & Solberg, S. Prediction of acute renal failure after cardiac surgery: retrospective cross - validation of a clinical algorithm. *Nephrology Dialysis Transplantation* **18**, 77-81 (2003).

46 Jorge-Monjas, P. *et al.* Predicting cardiac surgery–associated acute kidney injury: The CRATE score. *Journal of critical care* **31**, 130-138 (2016).

47 Slankamenac, K., Beck-Schimmer, B., Breitenstein, S., Puhan, M. A. & Clavien, P.-A. Novel prediction score including pre-and intraoperative parameters best predicts acute kidney injury after liver surgery. *World journal of surgery* **37**, 2618-2628 (2013).
